# Reaching a tipping point: Perioperative nurse managers’ narratives about reasons for leaving their employment—A qualitative study

**DOI:** 10.1111/jonm.13202

**Published:** 2020-11-24

**Authors:** Erebouni Arakelian, Gudrun Rudolfsson

**Affiliations:** ^1^ Department of Surgical Sciences Uppsala University Hospital Uppsala University Uppsala Sweden; ^2^ Faculty of Nursing and Health Sciences Nord University Bodø Norway; ^3^ Division of Nursing Department of Health Sciences University West Trollhättan Sweden

**Keywords:** dignity, leaving work, nurse manager, qualitative analysis

## Abstract

**Aim:**

To describe reasons why nurse managers in perioperative settings decide to leave their employment.

**Background:**

Current literature has shown that perioperative nurse managers’ reasons to leave their positions are formed through an interaction of factors.

**Methods:**

Individual in‐depth interviews were performed with seven nurse managers, all women, in perioperative settings in Sweden. Data were analysed using systematic text condensation.

**Results:**

Five key themes were identified: (a) to end where I started, as a frontline nurse; (b) I wanted to develop further to the next level in my career; (c) I ran out of ideas; (d) I lost trust in my head manager and did not believe in the new organisation and (e) I had had enough of being offended by my superior manager and my employees.

**Conclusion:**

Nurse managers experienced feeling forced into a decision to leave because of being offended by their superiors or their employees. Furthermore, the findings indicate that nurse managers should be offered support from superior managers and the organisation together with time for discussions.

**Implications in Nursing Management:**

The most essential element should be the influence of caritative leadership and the obvious expectation of being treated with dignity, respect and appreciation.

## 1 INTRODUCTION

Important cornerstones in a nurse's managerial role are foundational knowledge, role orientation, self‐development and mentorship (Moore et al., 2016). It is not unusual that nurse managers are recruited from within their own organisation, which means that they might not have the proper education or initial training for their tasks as nurse managers (Brown et al., 2013).

Perioperative nurse managers are crucial to the success of an operating department. There are many competencies that nurse managers should have in order to ensure organisational success (Gunawan et al., 2019). However, they risk being squeezed by demands for both quality of care and efficiency in the organisation. The organisation's ability to convey to the individual nurse manager, by congruence between actions and words, the sense that he/she is a valued employee, is dependent on the prevailing culture and value system (Brown et al., 2013). Parsons and Stonestreet (2003) highlight communication as the dominant factor that contributes to nurse managers’ retention, including accessibility of a superior for listening, guidance, clear expectations and feedback. Likewise, a study by Arakelian et al. (2020) demonstrated that nurse managers found their way and their inspiration as leaders through their employees’ strengths and motivation, and this was the driving force to stay. Saifman and Sherman (2019) put forward the link between the nurse manager's workload and role retention. In the same study, 44% of nurse managers who were responsible for more than one unit reported that they were either planning to leave or were uncertain whether they would stay.

It is crucial to examine how organisational, personal and position factors are related to each other and to determine which are more likely to lead to a decision to leave (Brown et al., 2013). Further, having reasonable workload expectations at all levels of organisational leadership was crucial for nurse managers when considering leaving (Hewko et al., 2015).

Brown et al. (2013) stress that the decision to leave is formed through an interaction of factors at organisational, managerial and personal levels. Other factors that influenced the nurse manager's decision to leave were, according to Hewko et al. (2015), lack of job satisfaction, lack of empowerment, an imbalance between work and leisure and the ability to ensure quality of care. Their findings also indicated the need for a trusting culture, which empowers leaders and understands the importance of acknowledging senior leaders whose responsibility is to prioritize the quality of patient care. They must support these nurse managers in their work. It is important for nurse managers not only to focus on developing other individuals but also to strengthen the conditions in which they can exercise their own leadership (Skagert et al., 2012).

In their study, Skytt et al. (2007) found that the dominant reason for nurse managers leaving their employment was re‐organisations and changes. The nurse manager's decision to leave was also affected by difficult situations in the relationship with the department head and his/her role performance, together with lack of support. In another study, Rudolfsson and Flensner (2012) found that perioperative nurse managers suffered when, despite their struggles, they failed to obtain support from their superiors and instead were questioned. They felt devalued, downhearted, ignored and as if in a void, which involved uncertainty and having limited options for action. This kind of suffering did not lead anywhere but encompassed feelings of discouragement, caused by mistrust, unfair criticism and humiliation. It seemed that their work had no purpose and they had no opportunity for learning and growth, factors that in the long term could lead them to leaving their nurse manager position (Rudolfsson & Flensner, 2012). That is why more information is needed about which managers leave and how to ensure that those managers who are needed most remain in their posts (Skagert et al., 2012).

Most published research in the context of perioperative nursing has focused on specialist nurses and their reasons for leaving their workplace (Logde et al., 2018) as well as on nurses in other contexts (Al Zamel et al., 2020).

Despite several searches for articles describing perspectives of perioperative nurse managers’ reasons for leaving their jobs, we have been unsuccessful in finding anything about this context, thus justifying this study.

### 1.1 Aim

The aim was to describe reasons why nurse managers in perioperative settings leave their employment.

## 2 METHODS

### 2.1 Design

Since our aim was to describe nurse managers’ experiences, a qualitative approach was undertaken in this study. Qualitative design is commonly used to study people's experiences of a phenomenon or a topic (Malterud, 2001, 2012, 2016). The study had a qualitative and prospective design and is a part of a larger research programme on nurse managers’ work environments (Arakelian et al., 2020). Individual interviews were carried out with seven perioperative nurse managers who had left their position during the last 6–12 months. According to the Consolidated Criteria for Reporting Qualitative Research (COREQ) checklist, we selected participants who shared specific characteristics and provided rich and diverse information (Tong et al., 2007).

### 2.2 The participants and settings

The inclusion criteria were as follows: nurse managers who had left their position in anaesthesia or operating department (perioperative settings) during the last 6–12 months. No exclusion criteria were set up. The human resources departments of the hospitals were contacted, to gain information about participants who met the inclusion criteria. Eleven nurse managers (all women) were invited to participate. To enable participants from different age groups and work experiences to be included, we used convenience sampling. The nurse managers were contacted through their work electronic mail and were informed about the study. An additional invitation was sent twice after the initial invitation if no answer was received. Informed consent was collected from those who agreed to participate, and an interview appointment was made. Seven nurse managers (five with a background as nurse anaesthetists and two with a background as operating room nurses), all women, agreed to participate in the study. One of the participants had a formal college education in leadership. The participants were between 53 and 61 years of age (mean age 55), and they had three to 18 years of experience as nurse managers. The participants were from three university hospitals and one county hospital in Sweden. The assignment to work as a nurse manager was a commitment for three years. Every three years, the two parties met to discuss whether the nurse manager wanted to stay or whether he/she had the head manager's confidence, or whether he/she should leave his/her position. The contract was renewed if the two parties entered a new agreement.

### 2.3 Data collection

An interview guide was designed for this study and included one main area, namely the reasons for leaving one's work. Four face‐to‐face interviews were performed and three telephone interviews, due to the long distance to the participants’ workplaces and homes. The first interview provided guidance as to the questions’ relevance before continuing the subsequent interviews. The initial question focused on the nurse manager's description of why they had left their former employment, followed by their main reasons for their decision to leave. Additionally, the nurse managers were asked to describe situations in which thoughts about leaving their work began to grow, but also the decisive situation in which they finally decided to leave. These issues were clarified by probing questions such as: Please tell me more. Can you give examples? What do you mean? The interviews were conducted by the first author between December 2018 and November 2019 and were 46–65 min long (mean 55 min). We concluded that there were no differences in the content based on the two methods of data collection.

### 2.4 Data analysis

Our method of analysis was systematic text condensation (STC) by Malterud (2001, 2012). The STC analysis followed these steps: a—to grasp the whole picture, the verbatim transcribed interviews were read through several times searching for meanings and patterns that were deemed to be important for the issue under study; b—preliminary themes emerged from the text and meaning units were identified; that is, passages of text relevant to the topic under investigation were coded; c—the resulting codes were condensed; that is, similar codes were grouped together; and d—re‐contextualization was performed, meaning that the final themes were created and the content for these themes was formed. In the final step, the interviews were read through once more, this time with the themes in mind. No new information could be identified after five interviews. However, the remaining interviews were analysed to reduce the risk of overlooking important information. Both researchers conducted all the steps in the analysis independently and the final version is a result of several discussions between them.

### 2.5 Ethical considerations

The study followed local ethical guidelines and regulations (Centre for Research Ethics and Bioethics, 2018) and the Declaration of Helsinki regulations (World Medical Association, 2013). This study was approved by the regional ethics committee in Uppsala, Sweden (Dnr 2018/381).

## 3 FINDINGS

Eleven nurse managers (all women) were invited to participate, and of these, seven women participated (Table 1). Six themes were identified as follows: a—to end where I started, as a frontline nurse, b—I wanted to develop further to the next level in my career, c**—**I ran out of ideas, d—I lost trust in my head manager and did not believe in the organisation, e—I was offended by my superior managers and f—I had had enough.

**Table 1:** Demographic data of the included participants

Nurse manager	Age (years)	Years as nurse manager	Background	Responsible for *N* staff members	Hospital
1	61	10	Nurse anaesthetist	85	University hospital
2	59	12	Operating room nurse	30	University hospital
3	57	18	Nurse anaesthetist	40	University hospital
4	53	9	Operating room nurse	100	County hospital
5	48	3	Nurse anaesthetist	55	University hospital
6	49	3	Nurse anaesthetist	180	University hospital
7	59	11	Nurse anaesthetist	30	University hospital

### 3.1 To end where I started, as a frontline nurse

Participants explained that by the end of their career, when they were approaching retirement age, they started to wonder whether they would have the energy left to be nurse managers. As a nurse manager, they worked from early morning to late afternoon, even after all other employees had left the workplace. They also had to be available after working hours and on weekends.

… It is a self‐preservation strategy…I do not know how long I will have the energy… I have no desire to move away from being in clinical work. Because it is my profession to do clinical work…. (Nurse Manager 1)

At that point, participants stated that it was important to stay close to their primary profession (as a nurse anaesthetist, for example) and to be able to return to their professional roots. However, in order to go back to being a frontline nurse, they had to refresh their theoretical and clinical knowledge, which was described as stimulating. The participants explained that they wanted a calmer work life compared to that of being a nurse manager, and this was a step in that direction. It meant having responsibility only for the patient's care and not for the whole operating department. They wished to end their career working closely with patients as a specialist nurse within their profession again. Participants explained that they had planned from the very beginning, when they first accepted the job as a nurse manager, to spend their last working years nursing patients again.

…I want to remember my professional time with my patients again. I would like to go back, pull up my anaesthesia injections in syringes, have a good patient encounter, do a good job… I will try to learn everything again…. (Nurse Manager 1).

### 3.2 I wanted to develop further to the next level in my career

Participants expressed that they left their current employment because they wanted to develop in their leadership and managerial roles. When offered a job of their dreams, they began to think about leaving their current employment. Furthermore, there was a desire to work with the next level of management, not simply to manage.

I've been close to quitting (before)… I’ve wanted to be in this (new) position, doing these tasks…. (Nurse Manager 6)

One nurse manager identified the need for a project manager within the organisation. When that position became available, it was seen as another opportunity to develop in a role and direction other than in management.

…I thought it was fun to do something else… they wanted a project manager… I was offered a project job… I had told them quite early that there were so many projects going on at the clinic, with no‐one in overall control, that we should have a project manager…. (Nurse Manager 5)

### 3.3 I ran out of ideas

Participants explained that after being with the same group of employees, with the same problems, for several years, they ran out of innovative ideas. They also realized that their employees and the operating department deserved a new leader with new power and energy. Participants expressed tiredness and dissatisfaction with not having the power or energy to renew themselves, and doubts began to rise. Therefore, they indicated that it was time for someone else with new energy to take over and develop the organisation further.

… you come to a point where you feel that these problems with this staff group… I can't think of any new solutions now, I ran out of ideas… Now someone else has to take over the baton. I decided long ago (when to quit)… this department deserves a new leader, new power… ten years is enough… you come to a turning point…. (Nurse Manger 1)

Despite describing and understanding that “all departments stagnate after a while…” (Nurse Manager 4), the nurse managers put demands on themselves thinking that even after several years of working with the same employees, they should come up with new ideas. It was important to the nurse managers that work did not become too much of a routine. They also pointed out that after several years, they had become trusted as nurse managers, yet they still felt the pressure to be an innovator.

… one of the staff members told me, we will miss you when you quit … You quit when you are at the top of your game… you do the right thing… but when I try to implement new ideas, I feel tired…. (Nurse Manager 1)

### 3.4 I lost trust in my head manager and did not believe in the new organisation

Participants explained that a functioning cooperation between the head manager or head physician and the nurse manager was essential for them to continue working as a nurse manager. Functioning cooperation was based on mutual trust. When this trust was broken or lost, they saw no point in continuing to work as a nurse manager. When the nurse manager did not have the same confidence and trust in a new head manager, there was no reason to stay.

… I was told that my head manager's position didn't get extended … I asked to speak to the area manager… he didn't give any sensible reasons (for letting my head manger go) … I had no confidence in him (the area manager) …. the last straw was that he didn't dare be honest with me … that's what tipped the balance….. (Nurse Manager 4)

To continue working as a nurse manager, one had to believe in the organisation. With re‐organisations and changing goals, the nurse managers had to decide whether to support the new organisation or to leave, and some chose the latter.

We underwent re‐organization… I believed in what we were to do (the goal) but it became apparent that many of the doctors didn't support the change that was needed… and this was very late in the process. We had worked with this change for 1.5 years… then I started wondering “Am I the right person to lead the way?” because I don't believe in what we are going to do now …. (Nurse Manager 6)

### 3.5 I had had enough of being offended by my superior manager and my employees

Lack of support or time for discussions with their head manager was one reason nurse managers wanted to leave their employment. Usually, before the nurse manager's contract was extended (typically every three years), a meeting was held between the two parties, the nurse manager and the head manager. When this communication was missing, no agreement could be achieved. Instead, it felt like an enforced decision. Such an instance was when the decision not to extend the nurse manager's contract was announced in passing in front of other employees, which created feelings of being offended or exposed to bullying.

…you have communication with each other about whether you want to continue or if the head of the department wants… to replace you. There is nothing strange about that… I thought we would have this meeting together, but my head manager turned it against me, suggesting that it was I who didn't want to stay…. (Nurse Manager 2)

…in front of all the employees… the head physician undermined and contradicted me… this was a question of survival for me… I thought, it's not worth it (staying on) …. (Nurse Manager 3)

The decision to quit their job was also caused by conflicts, disagreements or contradicting the nurse manager's views on important questions when having meetings with the heads of other departments. It was described as crucial to be a united front to the “outside world”, and for the nurse manager and the head manger to have the same view of how the business should be managed. In other words, “to speak the same language”.

Participants explained that being a nurse manager meant a great amount of work, with responsibility for employees and for implementing decisions from department heads, which led to conflicts between the nurse managers and their employees. It was “no bed of roses to be a manager” (Nurse Manager 4). Employees sometimes accused the nurse managers of not appreciating them or understanding their work tasks, although the nurse managers believed the opposite. In a conflict situation, several attempts were made involving the head managers of the organisation to solve the misunderstandings between the nurse managers and their employees but without any effect. This made the nurse managers feel unfairly treated by their employees, which ultimately resulted in attitudes that disparaged and affected their employees.

I felt that I got really mad when all these little things came. I wasn't a good nurse manager … I felt that I had had enough for a while, I had to take a break… and get some distance. (Nurse Manager 5)

The nurse managers felt publicly humiliated when, for example, employees started to shout at them without showing any respect. The nurse managers explained that when they and their employees could not come to an agreement, then it was time for the nurse manager to leave.

There was a situation that degenerated when one of the employees shouted at me (in front of everyone) … no one said stop … I am an employee too… it was an abuse… I felt offended… enough is enough … I don't want this anymore…. It was not worth this resistance (from the employees), this disrespect… (Nurse Manager 7)

The nurse managers felt that they were expected to solve every problem and that their employees took no responsibility on their own. This drained them of all energy. Thoughts began to emerge as to whether it was worth continuing as a nurse manager. The employees’ actions were experienced as “high level bullying” (Nurse Manager 5), which made the nurse managers feel devastated, and led to the decision to leave their employment.

## 4 DISCUSSION

Participants gave several reasons for why they wanted to leave their employment as nurse managers. Those who left their job voluntarily described it as a more mature decision, namely to end their career where they had originally started, that is, as a frontline nurse. Other mature career decisions included the desire to develop further to the next level in one's career. Another reason was having come to the conclusion that one's ideas about leadership had dried up. Other reasons to leave were of a more serious nature and nurse managers felt decisions had been forced upon them, affecting them on a deeper level. These were as follows: losing trust in a superior manager and not believing in the new organisation, and no longer being able to tolerate had enough of being offended by the superior manager and their employees.

The findings in this study indicated that some of the reasons for nurse managers leaving their jobs were either to seek new opportunities and challenges for furthering their managerial career, or to end their career by reverting to being a nurse. To end their career by reverting to nursing duties (their original starting point) can even be regarded as a development because they must refresh old knowledge and also update themselves with advances in the nursing profession, as one of the participants alluded to (Nurse Manager 1).

The theme “when I ran out of ideas” represents a demand which the nurse managers placed on themselves, always wondering why and how they were not able to develop the organisation further. Although earlier studies (DeCampli et al., 2010; Saifman & Sherman, 2019) point out that nurse managers are important for organisational development, the nurse managers in this study were pressured from both their superiors and their employees. Nurse managers struggle with their identity and support is needed to strengthen that identity (Harvey et al., 2014). Support from superior managers is essential if a nurse manger is to continue working (Adriaenssens et al., 2017; Skytt et al., 2007; Titzer et al., 2013). Lack of support leads to suffering in nurse managers, creating feelings of uncertainty (Rudolfsson & Flensner, 2012).

Our findings also concerned lack of trust in the superior manager and organisation, and receiving disrespectful treatment not only from superior managers, or department heads, but also from one's own employees. These conditions caused the nurse managers distress and gave them the feeling of being completely alone in the situation. Causing nurse managers to suffer always implies a violation of dignity, leading to a lack of confirmation of their full worth as human beings (Hampton et al., 2019). Suffering generated by human evil has no meaning whatsoever and implies that the condition of health has been disturbed (Eriksson, 2006). Therefore, trust can be understood as an important quality of a good relationship between the nurse leaders and others around them (Caldwell & Dixon, 2010). According to Caldwell and Dixon (2010), a culture that embraces trust as its core value does not seek to create a perfect work environment, but an environment that inspires individuals to build relationships.

In this study, conflicts arose when superior managers did not agree with the nurse managers or when head physicians opposed them concerning important questions or discussions. Depending on the situation, nurse managers can either enter the struggle or give up (Eriksson, 2006). There were also conflicts, in meetings with other departments and department heads or in meetings with employees. In this study, it was considered crucial to the “outside world” that the nurse managers and the head managers held the same opinions and “spoke the same language”.

To create an environment of responsiveness and tolerance, it is the responsibility of the superior manager to provide time and room for reflection and ideas. According to Bondas (2003), humiliating the nurse managers will also result in humiliation of one's own dignity when one acts in violation of one's own inner purpose as derived from the theory of caritative leadership. This theory was laid out by Bondas (2003) by means of theoretical deduction, exploring and analysing theories and concepts pertaining to leadership and administration from a caring science perspective. The core of caritative leadership is the *caritas* concept of human love and mercy. This philosophy is informed by thoughts from Eriksson's theory of caritative caring (Eriksson, 1992) and refers to the word administration.

Perhaps the most noteworthy finding in this study was the feeling of being offended or “bullied” by one's superior managers or mistreated by one's employees. Workplace incivility and counterproductive work behaviour include disrespect, poor communication and abusive behaviour, harming both the organisation and its members (Abolfazl Vagharseyyedin, 2015; Meier & Semmer, 2013). This kind of mistreatment was caused by physicians or superior managers (Guidroz et al., 2010). Hampton et al. (2019) indicated that those who are being bullied leave the organisation because this is one of most common strategies to avoid it. Thus, it is important to have a respectful relationship with, and understanding between, the nurse managers, their employees and superior managers. As suggested by Näsman (2018), attention should be paid to the importance of the basic values of the leader and the organisation. This understanding affects the opportunity for caritative leadership, where nurse managers can feel that they are seen and acknowledged.

## 5 LIMITATIONS

This study is likely to be relevant to a relatively small part of the readership as it addresses a limited number of nurse managers’ (due to one year of data collection) situations in a perioperative context. Hopefully, it may still be of interest. Perhaps it was mostly those who had been treated unfairly who agreed to participate in the study and this may have caused a bias. However, our findings also included nurse managers who had left their employment for other reasons. It may be cautiously suggested that our findings could have implications for nurse managers in other settings and countries as well. Credibility was judged in relation to the extent to which the description of the findings was consistent with the selected quotations and themes (Malterud, 2001; Malterud et al., 2016). Furthermore, confirmation of credibility and transferability was provided by a thorough description of the different stages of the analysis process (Malterud, 2001). The risk of overlooking important information was reduced by reading the text several times. This also addresses how well the themes covered the data, thus enhancing the credibility of the findings. Only women agreed to participate although nurse managers of both genders were invited. Perhaps male nurse managers are less frequently subject to bullying and demeaning behaviour, simply because they are male. However, this mirrors reality since there are fewer male nurse managers than female.

The authors’ professional pre‐understanding of the perioperative context might have influenced the analysis process and therefore bias cannot be entirely ruled out. While professional pre‐understanding could lead to deeper content in the interviews, it also constitutes a risk that familiar facts may have been overlooked. By bearing this in mind during the research process, the issue was consciously addressed and discussed, thus strengthening the analysis and contributing new and valuable angles (Malterud, 2001).

## 6 CONCLUSION

To end a career by reverting to nursing duties can be regarded as a natural consequence of a long working life as a nurse manager. The nurse manager's experiences of being offended by their superiors or their employees forced their decision to leave. Furthermore, the findings indicate that nurse managers should be offered support from the superior managers and organisation, together with time for discussions. It was important to have a respectful relationship with, and understanding between, the nurse managers, their employees and superior managers. Perhaps the most essential element of all for retaining nurse managers should be the obvious expectation of being treated with dignity, respect and appreciation by their superior managers and their employees.

## 7 IMPLICATIONS FOR NURSING MANAGEMENT

The study findings have relevance to practice for nurse managers in perioperative settings as they bring to the fore the nurse managers’ reasons for leaving their positions. The most essential element of all should be the influence of caritative leadership and the obvious expectation of being treated with dignity, respect and appreciation and to better prepare the next generation of nurse managers for their managerial tasks. Hopefully, this will contribute to the retention of nurse managers.

## ETHICAL APPROVAL

The study followed local ethical guidelines and regulations (Centre for Research Ethics and Bioethics, 2018) and the Declaration of Helsinki regulations (World Medical Association, 2013). This study was approved by the regional ethics committee in Uppsala, Sweden (Dnr 2018/381).
